# Liraglutide Suppresses Obesity and Hyperglycemia Associated with Increases in Hepatic Fibroblast Growth Factor 21 Production in KKA^y^ Mice

**DOI:** 10.1155/2014/751930

**Published:** 2014-04-07

**Authors:** Katsunori Nonogaki, Miki Hazama, Noriko Satoh

**Affiliations:** Department of Lifestyle Medicine, Translational Research Center, Tohoku University Hospital, 1-1 Seiryo-machi, Aoba-ku, Sendai, Miyagi 980-8574, Japan

## Abstract

Social isolation contributes to the development of obesity and insulin-independent diabetes in KKA^y^ mice. Here we show that systemic administration of liraglutide, a long-acting human glucagon-like peptide-1 (GLP-1) analog, significantly decreased food intake, body weight, and blood glucose levels at 24 h after its administration while having no significant effects on plasma insulin and glucagon levels in individually housed KKA^y^ mice. In addition, the systemic administration of liraglutide significantly increased plasma fibroblast growth factor (Fgf) 21 levels (1.8-fold increase) associated with increases in the expression of hepatic *Fgf21* (1.9-fold increase) and *Pparγ* (1.8-fold increase), while having no effects on the expression of hepatic *Pparα* and *Fgf21* in white adipose tissue. Moreover, systemic administration of liraglutide over 3 days significantly suppressed food intake, body weight gain, and hyperglycemia in KKA^y^ mice. On the other hand, despite remarkably increased plasma active GLP-1 levels (4.2-fold increase), the ingestion of alogliptin, a selective dipeptidyl peptidase-4 inhibitor, over 3 days had no effects on food intake, body weight, blood glucose levels, and plasma Fgf21 levels in KKA^y^ mice. These findings suggest that systemic administration of liraglutide induces hepatic Fgf21 production and suppresses the social isolation-induced obesity and diabetes independently of insulin, glucagon, and active GLP-1 in KKA^y^ mice.

## 1. Introduction

We have previously reported that social isolation contributes to the development of obesity and type 2 diabetes [1]. In KKA^y^ mice with ectopic overexpression of agouti peptide, an endogenous melanocortin-4 receptor (MC4R) antagonist, social isolation promotes obesity due to the primary decreased energy expenditure and secondary increased food consumption [1]. In addition, social isolation leads to the insulin-independent diabetes associated with increased expression of hepatic gluconeogenic genes in KKA^y^ mice [1]. The therapeutic agents for the social isolation-induced obesity and diabetes, however, remain uncertain.

Glucagon-like peptide-1 (GLP-1) is an incretin hormone that is released from intestinal L-cells in response to nutrient ingestion [2]. GLP-1 potentiates glucose-dependent insulin secretion by activating the GLP-1Rs that are expressed on pancreatic islet *β*-cells [2]. Liraglutide, a human GLP-1 analog, is a novel, long-acting GLP-1 derivative that is resistant to dipeptidyl peptidase-4 (DPP-4) [3], which rapidly degrades from the active form of GLP-1 (7–36) to an inactive, *N*-terminally truncated form (9–36) in the bloodstream. Its prolonged effects result from the substitution of Lys for Arg34 and the addition of a glutamic acid and a 16C fatty acid chain to the Lys26 residue of native GLP-1 [3]. DPP-4 inhibitors have emerged as a new class of agents demonstrated to improve glycemic control, principally by potentiating the action of endogenously secreted incretin [4, 5]. Alogliptin is a highly selective, quinazolinone-based, noncovalent DPP-4 inhibitor as a once-daily treatment for type 2 diabetes [6–8].

To determine the effects of liraglutide on obesity and insulin-independent diabetes in individually housed KKA^y^ mice, we measured food intake, body weight change, blood glucose, plasma insulin, and fibroblast growth factor (Fgf) 21 levels and the expression of hepatic proliferator-activated receptor *α* (*Ppar*α**),* Ppar*γ**, glucose-6-phosphatase (*G6pase*), forkhead box protein O1 (*Foxo1*), and* Fgf21*, which are involved in the regulation of the glucose metabolism, and the expression of* Fgf21* in epididymal white adipose tissue in individually housed KKA^y^ mice 24 h after an intraperitoneal injection of liraglutide. In addition, we examined the effects of systemic administration of liraglutide for 3 days on the development of obesity and diabetes in individually housed KKA^y^ mice.

To determine the role of active GLP-1 in plasma on the social isolation-induced obesity and type 2 diabetes, we examined the effects of the ingestion of alogliptin for 3 days on daily food intake, body weight gain, blood glucose levels, and plasma active GLP-1 and Fgf21 levels in individually housed KKA^y^ mice.

## 2. Materials and Methods 

Four-week-old male KKA^y^ mice were purchased from Japan CLEA. The mice were group housed in cages with free access to water and chow pellets in a light- and temperature-controlled environment (12 h on/12 h off, lights on at 08:00 and lights off at 20:00; 20–22°C). One week later, the mice were housed in individual cages with free access to water and a fish meal-free diet (fish meal-free F1: 4.4% fat; Funabashi Farm, Funabashi, Japan) on a 12 h light-dark cycle (lights off at 20:00 hours) in a temperature-controlled (20–22°C) environment.

In the first experiment, 6-week-old male KKA^y^ mice were then intraperitoneally injected with saline or liraglutide (150 *μ*g/kg). The animals were fed the fish meal-free pellets after being treated. 24 h later, food intake and body weight were measured. Then, the animals were decapitated, and the blood was collected for the measurements of blood glucose and plasma insulin and Fgf21. Mean daily food consumption per day and body weight changes were measured.

In the second experiment, 6-week-old male KKA^y^ mice were then intraperitoneally injected with saline or liraglutide (150 *μ*g/kg) once a day for 3 days. At the end of the third day, the animals were decapitated, and the blood was collected for the measurements of blood glucose levels. Mean daily food consumption per day and body weight changes were measured.

In the third experiment, 6-week-old male KKA^y^ mice were provided a fish meal-free diet with or without alogliptin (0.03%) for 3 days. At the end of the third day, the animals were decapitated, and the blood was collected for the measurements of blood glucose and plasma active GLP-1 and plasma Fgf21. Mean daily food consumption per day and body weight changes were measured.

The dose of liraglutide (150 *μ*g/kg) was selected based on evidence that liraglutide induces hypophagia in mice [9]. Liraglutide was a kind gift from Novo Nordisk, Japan. The drugs were dissolved in 0.2 mL 0.9% saline. The doses of alogliptin were used as described previously [7, 8]. Alogliptin was a kind gift from Takeda Pharmaceutical Co, Japan. Blood glucose levels were measured using glucose strips (blood glucose monitoring system; FreeStyle, NIPRO, Tokyo, Japan). The experiment was performed at between 10:00 and 12:00.

The whole blood was mixed with EDTA-2Na (2 mg/mL) and aprotinin (500 kIU/mL) to determine the plasma levels of insulin, active GLP-1, and Fgf21. Plasma levels of active GLP-1 were measured by an enzyme-linked immunosorbent assay (mouse active GLP-1 ELISA kit; Shibayagi Inc., Gunma, Japan) as described previously [10, 11]. The plasma levels of Fgf21 were measured by ELISA (rat/mouse Fgf21 ELISA kits; R&D system, Tokyo, Japan). The plasma levels of insulin were measured by radioimmunoassay (rat insulin RIA kit; Millipore Corporation, USA). The levels of glucagon were measured by double-antibody radioimmunoassay (glucagon RIA kit (SML); Euro-Diagnostica AB, Sweden). The animal studies were conducted in accordance with the institutional guidelines for animal experiments at the Tohoku University Graduate School of Medicine.

Data are presented as the mean ± SEM (*n* = 6). The comparisons between two groups were performed with Student's* t*-test. A* P* value of less than 0.05 was considered to be statistically significant.

### 2.1. Real-Time Quantitative RT-PCR

Total RNA was isolated from mouse liver using the RNeasy Midi kit (Qiagen, Hilden, Germany) and epididymal white adipose tissue (eWAT) using the RNeasy Lipid Tissue Midi kit (Qiagen, Hilden, Germany) according to the manufacturer's directions. cDNA synthesis was performed using a Super Script III First-Strand Synthesis System for RT-PCR Kit (Invitrogen, Rockville, MD) using 1 *μ*g total RNA. cDNA synthesized from total RNA was evaluated in a real-time PCR quantitative system (LightCycler Nano Instrument Roche Diagnostics, Mannheim, Germany). The primers used are listed in Table 1. The relative amount of mRNA was calculated using *β*-actin mRNA as the invariant control. The data are shown as the fold change of the mean value of the control group, which received saline as described previously [1].

## 3. Results

### 3.1. Effects of Liraglutide on Food Intake, Body Weight, Blood Glucose, Plasma Insulin, Glucagon, and Fgf21 Levels in *KKA* ^*y*^ Mice

**Figure 1:**
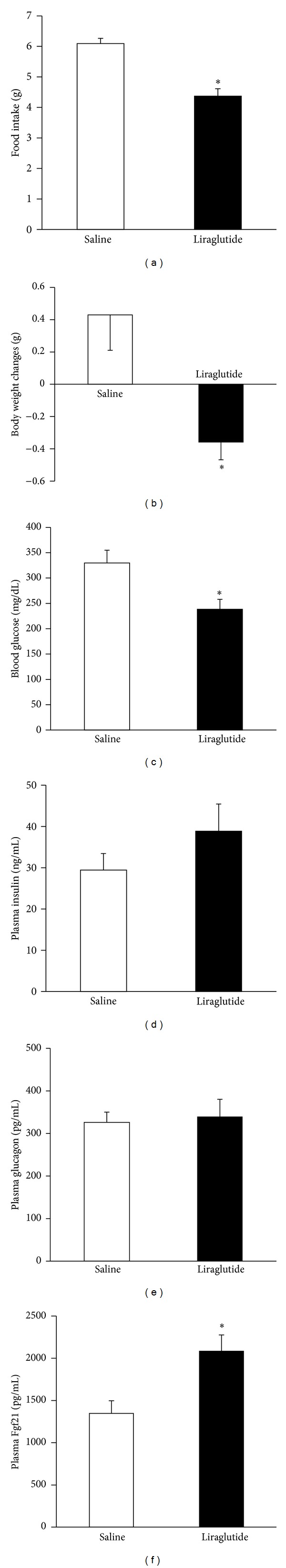
The effects of intraperitoneal injection of liraglutide (150 *μ*g/kg) or saline on (a) food intake, (b) body weight, (c) blood glucose levels, (d) plasma insulin, (e) glucagon, and (f) Fgf21 levels in individually housed KKA^y^ mice are determined 24 h after treatment, as described in the Materials and Methods section. Basal body weight in 6-week-old KKA^y^ mice treated with or without liraglutide was 33.0 ± 0.6 g and 33.1 ± 0.5 g, respectively. The data are presented as the mean ± SEM (*n* = 6 for each group). *P < 0.05.

In 6-week-old KKA^y^ mice, the intraperitoneal injection of liraglutide (150 *μ*g/kg) significantly decreased food intake (Figure 1(a)), body weight (Figure 1(b)), and blood glucose levels (Figure 1(c)) and increased plasma levels of Fgf21 (1.8-fold) at 24 h compared with the saline control (Figure 1(f)), while having no significant changes in plasma insulin (Figure 1(d)) and glucagon levels (Figure 1(e)). These findings suggest that systemic administration of liraglutide reduces hyperphagia, obesity, and hyperglycemia associated with increased Fgf21 levels in plasma.

### 3.2. Effects of Liraglutide on the Expression of Hepatic Fgf21, Ppar*γ*, Ppar*α*, G6pase, and Foxo1 in *KKA* ^*y*^ Mice

**Figure 2:**
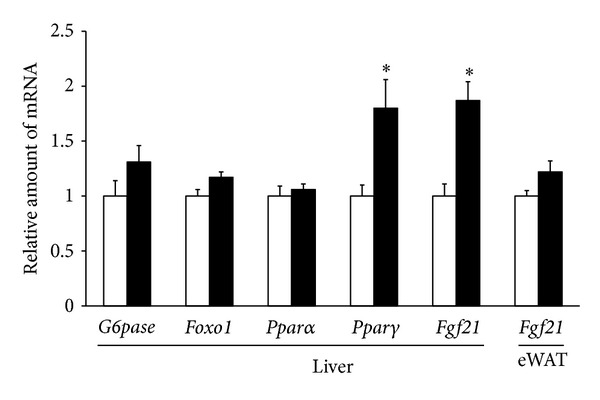
The effects of intraperitoneal injection of liraglutide (150 *μ*g/kg) or saline on the expression of hepatic* Ppar*γ*, Ppar*α*, G6pase, Foxo1,* and* Fgf21* and* Fgf21* in epididymal white adipose tissue (eWAT) in individually housed KKA^y^ mice are determined 24 h after liraglutide treatment, as described in the Materials and Methods section. The data are presented as the mean ± SEM (*n* = 6 for each group). *P < 0.05.

In 6-week-old KKA^y^ mice, the intraperitoneal injection of liraglutide (150 *μ*g/kg) also significantly increased the expression of hepatic* Ppar*γ** (1.8-fold increase) and* Fgf21* (1.9-fold increase) while having no significant changes in the expression of* Ppar*α**,* G6pase*, and* Foxo1* in the liver and the expression of* Fgf21* in epididymal white adipose tissue at 24 h compared with the saline control (Figure 2).

These findings suggest that systemic administration of liraglutide increased expression of hepatic* Fgf21* and* Ppar*γ*,* while having no effects on hepatic gene expression involved in the regulation of hepatic glucose production.

### 3.3. Effects of Ingestion of Alogliptin or Systemic Administration of Liraglutide for 3 Days on Obesity and Insulin-Independent Diabetes in *KKA* ^*y*^ Mice

**Figure 3:**
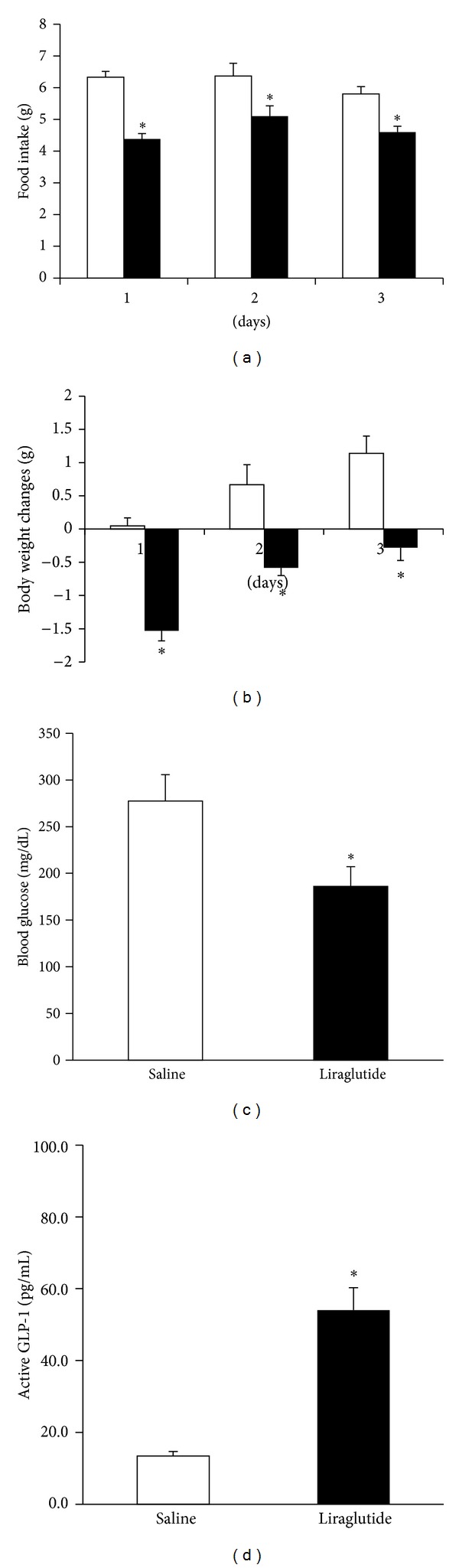
The effects of intraperitoneal injection of liraglutide (150 *μ*g/kg) or saline over 3 days on (a) daily food intake, (b) body weight changes, (c) blood glucose levels, and (d) plasma active GLP-1 levels in individually housed KKA^y^ mice. Open bar; saline controls and filled bar; liraglutide treatment. Basal body weight in 6-week-old KKA^y^ mice treated with or without liraglutide was 31.2 ± 0.6 g and 32.2 ± 0.5 g, respectively. Data are presented as the mean values ± SEM (*n* = 6 for each group of animals). *P < 0.05.

In 6-week-old KKA^y^ mice, the intraperitoneal injection of liraglutide (150 *μ*g/kg) over 3 days significantly decreased daily food intake (Figure 3(a)), body weight gain (Figure 3(b)), and blood glucose levels (Figure 3(c)) and significantly increased plasma active GLP-1 levels (Figure 3(d)) compared with saline controls.

**Figure 4:**
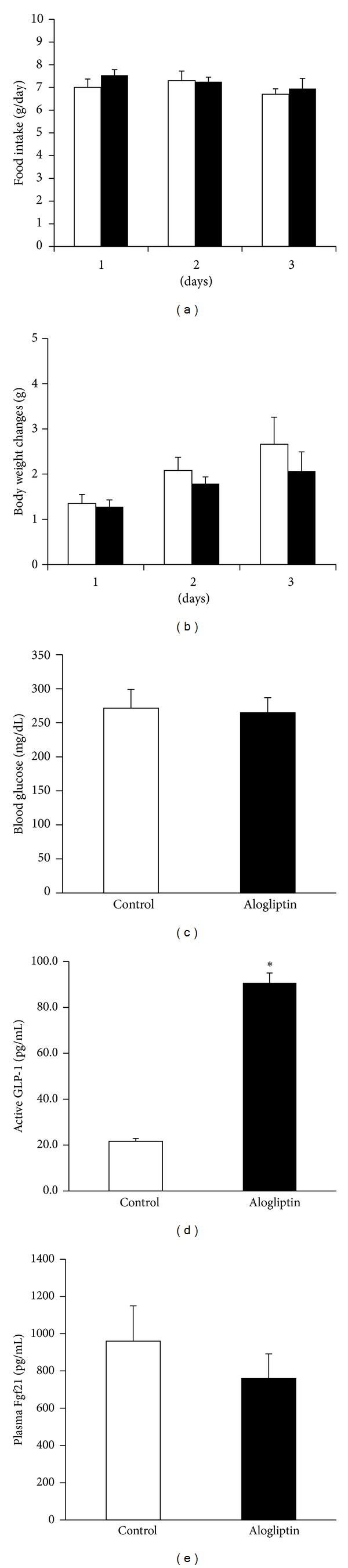
The effects of ingestion of a fish meal-free diet with or without alogliptin (0.03%) over 3 days on (a) daily food intake, (b) body weight changes, (c) blood glucose levels, (d) plasma active GLP-1, and (e) Fgf21 levels in individually housed KKA^y^ mice. Open bar; a fish meal-free diet without alogliptin and filled bar; a fish meal-free diet with alogliptin (0.03%). Basal body weight in 6-week-old KKA^y^ mice treated with or without alogliptin was 32.7 ± 0.9 g and 33.3 ± 0.5 g, respectively. Data are presented as the mean values ± SEM (*n* = 6 for each group of animals). *P < 0.05.

On the other hand, the ingestion of a fish meal-free diet with alogliptin over 3 days had no effect on daily food intake (Figure 4(a)), body weight gain (Figure 4(b)), blood glucose levels (Figure 4(c)), or plasma Fgf21 levels (Figure 4(e)), although alogliptin remarkably increased plasma active GLP-1 levels (4.2-fold increase) compared with controls (Figure 4(d)).

These findings suggest that the treatment with alogliptin has no effects on obesity, hyperglycemia, and plasma Fgf21 levels in individually housed KKA^y^ mice, whereas the treatment with liraglutide reduces the obesity and hyperglycemia independently of plasma active GLP-1 levels.

## 4. Discussion

We previously reported that systemic administration of liraglutide suppresses food intake and body weight in mice with a serotonin 5-HT2C receptor (5-HT2CR) null mutation and heterozygous melanocortin-4 receptor (MC4R) mutation, suggesting that functional 5-HT2CR and MC4R pathways are not essential for the inhibitory effects of liraglutide on food intake and body weight in mice [9]. The present study supported our previous findings and suggests that liraglutide exerts the suppressive effects on hyperphagia, obesity, and hyperglycemia induced by the perturbed central MC4R signaling. In addition, the present study demonstrated that liraglutide had no significant effects on the expression of hepatic* G6pase* and* Foxo1* which are involved in hepatic glucose production. The inhibitory effects of liraglutide on hyperglycemia may therefore be due to the increased glucose uptake in the peripheral tissues but not suppressing hepatic glucose production in KKA^y^ mice.

Fgf21 is an atypical member of the Fgf family that functions as an endocrine hormone to regulate glucose and lipid metabolism [12]. When administered pharmacologically to obese and insulin resistant rodents, Fgf21 increases energy expenditure, insulin sensitivity, and weight loss and normalizes glucose and lipid levels [12–15]. The increase in circulating Fgf21 induced by liraglutide might therefore contribute to the improvement of obesity and hyperglycemia in individually housed KKA^y^ mice. Yang et al. reported that chronic administration of high dose liraglutide (1 mg/kg) twice daily for 8 weeks increased plasma Fgf21 levels and improved insulin resistance in high fat diet-fed mice with ApoE deficiency and hypoadiponectinemia [16]. The present study supports the previous evidence and demonstrated that the lower dose liraglutide increased hepatic Fgf21 production within 24 h.

In mice, Fgf21 is increased in liver by fasting through a mechanism that requires the nuclear fatty acid receptor,* Ppar*α** [17, 18]. During fasting,* Fgf21* expression in liver is controlled by* Ppar*α**, and pharmacologic administration of* Ppar*α** agonists increases the expression of hepatic* Fgf21* [17, 18]. The present study, however, demonstrated that increased expression of hepatic* Fgf21* induced by liraglutide was not associated with the increased expression of hepatic* Ppar*α**. Thus, liraglutide may induce hepatic Fgf21 production via a different pathway than food deprivation and/or* Ppar*α* in vivo*.

In addition, Fgf21 is induced by* Ppar*γ** agonists, including thiazolidinediones, in white adipose tissue [19], and regulates the activity of* Ppar*γ**, a master transcriptional regulator of adipogenesis [20]. Fgf21 is therefore suggested as a key mediator of the physiologic and pharmacologic actions of* Ppar*γ** [20]. The present study demonstrated that liraglutide increased the expression of hepatic* Ppar*γ** as well as* Fgf21 *while having no effects on the expression of* Fgf21* in epididymal white adipose tissue, suggesting that the increases in plasma* Fgf21* levels induced by liraglutide are due to the increased hepatic* Fgf21* production associated with increased* Ppar*γ** activation.

Moreover, Fgf21 is suggested to mediate some metabolic actions of glucagon. Native glucagon increases plasma Fgf21 levels in human subjects [21], and a synthetic glucagon receptor agonist (IUB288) upregulates* Fgf21* expression in isolated primary hepatocytes from mice [22]. Although GLP-1 reportedly increases insulin secretion and suppresses glucagon secretion [23], the results of the present study demonstrated that despite the reduction of hyperglycemia, systemic administration of liraglutide had no effects on plasma insulin and glucagon levels in KKA^y^ mice. The unchanged insulin was seen in the presence of a lower glucose, suggesting that liraglutide stimulates *β*-cell function and works through stabilizing insulin levels in spite of lower glucose. The unchanged glucagon was seen in the presence of a lower glucose, suggesting that liraglutide suppresses *α*-cell function and works through stabilizing glucagon levels, although glucagon unlikely contributes to the liraglutide-induced increase in hepatic Fgf21 production and suppression of hyperglycemia in KKA^y^ mice.

It remains uncertain whether liraglutide directly induces hepatic Fgf21 production* in vivo*. The present results demonstrated that increased active GLP-1 in plasma induced by the treatment with alogliptin had no effects on plasma levels of Fgf21. Alogliptin has no effects on hyperglycemia in db/db mice with a leptin receptor mutation [8]. Moreover, the present findings demonstrated that despite elevated plasma levels of active GLP-1, treatment with alogliptin did not suppress hyperglycemia in individually housed KKA^y^ mice. Thus, insulin-independent diabetes associated with obesity, which have perturbed leptin receptor and/or MC4R signaling, may be resistant to the DPP-4 inhibitor. Because GLP-1Rs are little expressed in the liver [23], the effects of liraglutide on hepatic Fgf21 production may be exerted via the central nervous system-mediated efferent pathways. Additional studies are needed to gain a better understanding of the mechanisms by which liraglutide induces hepatic Fgf21 production and the role of Fgf21 in social isolation-induced diabetes in KKA^y^ mice.

## 5. Conclusions 

These findings suggest that systemic administration of liraglutide induces hepatic Fgf21 production and suppresses the social isolation-induced development of obesity and hyperglycemia independently of insulin, glucagon, and active GLP-1 in KKA^y^ mice.

## Figures and Tables

**Table 1:** The primer used for real-time RT-PCR.

Gene	Primer	Sequence
*Ppar*α**	Sense	CGGGTAACCTCGAAGTCTGA
	Antisense	CTAACCTTGGGCCACACCT

*Ppar*γ**	Sense	CTGCTCAAGTATGGTGTCCATGAG
	Antisense	GAGGAACTCCCTGGTCATGAATC

*G6pase *	Sense	TGCAAGGGAGAACTCAGCAA
	Antisense	GGACCAAGGAAGCCACAATG

*Foxo1 *	Sense	GCGTGCCCTACTTCAAGGATAA
	Antisense	TCCAGTTCCTTCATTCTGCACT

*Fgf21 *	Sense	CGCAGTCCAGAAAGTCTCC
	Antisense	ATCAAAGTGAGGCGATCCA

## References

[1] Nonogaki K, Nozue K, Oka Y (2007). Social isolation affects the development of obesity and type 2 diabetes in mice. *Endocrinology*.

[2] Holst JJ (2007). The physiology of glucagon-like peptide 1. *Physiological Reviews*.

[3] Drucker DJ, Dritselis A, Kirkpatrick P (2010). Liraglutide. *Nature Reviews Drug Discovery*.

[4] Lovshin JA, Drucker DJ (2009). Incretin-based therapies for type 2 diabetes mellitus. *Nature Reviews Endocrinology*.

[5] Feng J, Zhang Z, Wallace MB (2007). Discovery of alogliptin: a potent, selective, bioavailable, and efficacious inhibitor of dipeptidyl peptidase IV. *Journal of Medicinal Chemistry*.

[6] Lee B, Shi L, Kassel DB, Asakawa T, Takeuchi K, Christopher RJ (2008). Pharmacokinetic, pharmacodynamic, and efficacy profiles of alogliptin, a novel inhibitor of dipeptidyl peptidase-4, in rats, dogs, and monkeys. *European Journal of Pharmacology*.

[7] Moritoh Y, Takeuchi K, Asakawa T, Kataoka O, Odaka H (2009). The dipeptidyl peptidase-4 inhibitor alogliptin in combination with pioglitazone improves glycemic control, lipid profiles, and increases pancreatic insulin content in *ob/ob* mice. *European Journal of Pharmacology*.

[8] Moritoh Y, Takeuchi K, Asakawa T, Kataoka O, Odaka H (2009). Combining a dipeptidyl peptidase-4 inhibitor, alogliptin, with pioglitazone improves glycaemic control, lipid profiles and *β*-cell function in *db/db* mice. *British Journal of Pharmacology*.

[9] Nonogaki K, Suzuki M, Sanuki M, Wakameda M, Tamari T (2011). The contribution of serotonin 5-HT2C and melanocortin-4 receptors to the satiety signaling of glucagon-like peptide 1 and liragultide, a glucagon-like peptide 1 receptor agonist, in mice. *Biochemical and Biophysical Research Communications*.

[10] Nagamatsu S, Ohara-Imaizumi M, Nakamichi Y, Aoyagi K, Nishiwaki C (2011). DPP-4 inhibitor des-F-sitagliptin treatment increased insulin exocytosis from *db/db* mice *β* cells. *Biochemical and Biophysical Research Communications*.

[11] Nonogaki K, Suzuki M (2013). Liraglutide suppresses the plasma levels of active and des-acyl ghrelin independently of active glucagon-like peptide-1 levels in mice. *ISRN Endocrinology*.

[12] Kharitonenkov A, Shiyanova TL, Koester A (2005). FGF-21 as a novel metabolic regulator. *The Journal of Clinical Investigation*.

[13] Berglund ED, Li CY, Bina HA (2009). Fibroblast growth factor 21 controls glycemia via regulation of hepatic glucose flux and insulin sensitivity. *Endocrinology*.

[14] Coskun T, Bina HA, Schneider MA (2008). Fibroblast growth factor 21 corrects obesity in mice. *Endocrinology*.

[15] Xu J, Lloyd DJ, Hale C (2009). Fibroblast growth factor 21 reverses hepatic steatosis, increases energy expenditure, and improves insulin sensitivity in diet-induced obese mice. *Diabetes*.

[16] Yang M, Zhang L, Wang C (2012). Liraglutide increases FGF-21 activity and insulin sensitivity in high fat diet and adiponectin knockdown induced insulin resistance. *PLoS ONE*.

[17] Inagaki T, Lin VY, Goetz R, Mohammadi M, Mangelsdorf DJ, Kliewer SA (2008). Inhibition of growth hormone signaling by the fasting-induced hormone FGF21. *Cell Metabolism*.

[18] Badman MK, Koester A, Flier JS, Kharitonenkov A, Maratos-Flier E (2009). Fibroblast growth factor 21-deficient mice demonstrate impaired adaptation to ketosis. *Endocrinology*.

[19] Muise ES, Azzolina B, Kuo DW (2008). Adipose fibroblast growth factor 21 is up-regulated by peroxisome proliferator-activated receptor *γ* and altered metabolic states. *Molecular Pharmacology*.

[20] Dutchak PA, Katafuchi T, Bookout AL (2012). Fibroblast growth factor-21 regulates PPAR*γ* activity and the antidiabetic actions of thiazolidinediones. *Cell*.

[21] Habegger KM, Stemmer K, Cheng C (2013). Fibroblast growth factor 21 mediates specific glucagon actions. *Diabetes*.

[22] Arafat AM, Kaczmarek P, Skrzypski M (2013). Glucagon increases circulating fibroblast growth factor 21 independently of endogenous insulin levels: a novel mechanism of glucagon-stimulated lipolysis?. *Diabetologia*.

[23] Campbell JE, Drucker DJ (2013). Pharmacology, physiology, and mechanisms of incretin hormone action. *Cell Metabolism*.

